# Targeting Features of Curaxin CBL0137 on Hematological Malignancies In Vitro and In Vivo

**DOI:** 10.3390/biomedicines11010230

**Published:** 2023-01-16

**Authors:** Timur I. Fetisov, Anna A. Borunova, Alina S. Antipova, Elena E. Antoshina, Lubov S. Trukhanova, Tatyana G. Gorkova, Svetlana N. Zuevskaya, Alexei Maslov, Katerina Gurova, Andrei Gudkov, Ekaterina A. Lesovaya, Gennady A. Belitsky, Marianna G. Yakubovskaya, Kirill I. Kirsanov

**Affiliations:** 1 N.N. Blokhin National Medical Research Center of Oncology, 115478 Moscow, Russia; 2 Department of Infectious Diseases, Sechenov University, 119991 Moscow, Russia; 3 Department of Cell Stress Biology, Roswell Park Comprehensive Cancer Center, Buffalo, NY 14263, USA; 4 Department of Oncology, I.P. Pavlov Ryazan State Medical University, 390026 Ryazan, Russia

**Keywords:** DNA-binding small molecules, minor groove binding DNA ligands, Curaxin CBL0137, anticancer epigenetic drug, cytotoxicity, signaling pathways, leukemias, multiple myeloma, mechanisms of antitumor activity, chemotherapy drugs

## Abstract

The anticancer activity of Curaxin CBL0137, a DNA-binding small molecule with chromatin remodulating effect, has been demonstrated in different cancers. Herein, a comparative evaluation of CBL0137 activity was performed in respect to acute myeloid leukemia (AML), acute lymphoblastic leukemia (ALL), chronic myeloid leukemia and multiple myeloma (MM) cultured in vitro. MTT assay showed AML and MM higher sensitivity to CBL0137’s cytostatic effect comparatively to other hematological malignancy cells. Flow cytometry cell cycle analysis revealed an increase in subG1 and G2/M populations after CBL0137 cell treatment, but the prevalent type of arrest varied. Apoptosis activation by CBL0137 measured by Annexin-V/PI dual staining was more active in AML and MM cells. RT2 PCR array showed that changes caused by CBL0137 in signaling pathways involved in cancer pathogenesis were more intensive in AML and MM cells. On the murine model of AML WEHI-3, CBL0137 showed significant anticancer effects in vivo, which were evaluated by corresponding changes in spleen and liver. Thus, more pronounced anticancer effects of CBL0137 in vitro were observed in respect to AML and MM. Experiments in vivo also indicated the perspective of CBL0137 use for AML treatment. This in accordance with the frontline treatment approach in AML using epigenetic drugs.

## 1. Introduction

Curaxin CBL0137 (CBL0137) is a carbazole derivative, characterized by a wide range of anticancer activities [1]. Chemical modification of DNA does not occur during CBL0137 interaction with the biopolymer, which explains the absence of genotoxic activity for this drug [2]. It interacts with DNA in a mixed manner, both intercalating between base pairs and binding to DNA grooves noncovalently. It changes the physicochemical and spatial characteristics of the DNA duplex, and influences functions of DNA-mediated proteins [3]. In particular, it inhibits protein complex FACT [2], a histone chaperone consisting of two subunits: SSRP-1 and SPT16. Not interacting with FACT directly, CBL0137 causes its redistribution in the cell nucleus. This is probably due to the influence of CBL0137 on DNA interaction with histone dimers H2A/H2B and H3/H4 [3]. CBL0137 has recently been shown to modulate the activity of enzymes involved in epigenetic regulation of gene expression; in particular, it inhibits the methyltransferase Dnmt3a, and it may explain the activation of retrotrasposone expression followed by interferon type I signaling [4,5]. CBL0137 influences the function of DNA-binding proteins involved in chromatin organization, in particular, its intercalation into DNA results in the inability of CTCF to bind to the corresponding cognate genomic sites [6,7]. Accordingly, CBL0137 can mediate disruption of the structure and boundaries of topologically associated domains (TAD), preventing efficient interaction between enhancers and gene promoters [7]. The observed effects of CBL0137 lead to significant transcriptome changes, in particular changes in the p53, NF-kB, IFN, NOTCH, and WNT/β-catenin [2,4,8] signaling pathways. Thus, CBL0137 is considered to remodulate chromatin, demonstrating epigenetic activity.

CBL0137’s anticancer activity has been revealed in respect to the number of solid tumors, including breast, pancreatic, colon, lung, skin neoplasms and brain tumors [1,8,9,10]. CBL0137’s antitumor effect was also demonstrated by the Pediatric Pre-Clinical Testing Program for juvenile forms of malignant neoplasms of the blood system on xenografts of highly aggressive mixed leukemia with alterations of the gene MLL/KMT2A and in vitro on T-cell acute lymphoblastic leukemia (ALL) COG-LL-317 [11,12]. Taking into consideration that the most intensive changes of epigenetic regulation among different cancers are observed in AML, and that epigenetic antitumor drugs were introduced in the treatment of AML, the aim of this study was to compare the antitumor activity of chromatin remodulator CBL0137 in vitro in respect to different hematological malignancies and to estimate CBL0137 effects in vivo using the mouse model of acute myeloid leukemia WEHI-3 [13,14]. 

## 2. Materials and Methods

### 2.1. Cell Lines 

Human AML cell lines THP-1 and KG-1, human ALL cell line CCRF-SB and murine AML cell line WEHI-3 were obtained from the collection of vertebrate cultures of the Institute of Cytology, Russian Academy of Sciences (St. Petersburg, Russia); human ALL cell line CCRF-CEM was kindly provided by Irina Budunova (Northwestern University, Chicago, IL, USA); human MM cell lines NCI-H929 and RPMI-8226 and CML cell line K562 were kindly provided by Natalia Moiseeva (N.N. Blokhin NMRCO, Moscow, Russia). Cells were used for no longer than 20 passages. Mycoplasma cell culture contamination was routinely checked using DAPI staining, followed by fluorescent microscopy. Cells were cultured in RPMI-1640 supplemented with 10% FSS and 20% FBS for KG-1 and 1% penicillin–streptomycin (Paneco LTD, RF) in a humidified incubator at 37 °C, 5% CO_2_.

Human blood samples were obtained from AML and ALL patients who had been treated at the N.N. Blokhin National Medical Research Center of Oncology. The Ethics Committee of N.N. Blokhin NMRCO approved the project, and all patients who were involved in the study gave written informed consent that their samples could be used for research purposes. The study protocol is in accordance with the ethical guidelines of the 1975 Declaration of Helsinki. Data were analyzed anonymously. Peripheral blood from leukemia patients was mixed 1:2 with RPMI 1640, overlaid on Ficoll-Paque (GE Healthcare, Pittsburgh, PA, USA) and centrifuged (600× *g*, 25 min). The PBMC layer was collected and cells washed twice by centrifugation (200× *g*, 8 min), followed by resuspension in RPMI 1640.

### 2.2. Cytotoxicity Analysis

Cell cultures were seeded into 96-well plates and incubated overnight under 5% CO2 at 37 °C for 24 h. Then cells were treated with CBL0137 (10–0.1 μM) for 24, 48 and 72 h. Then, 20 μL MTT solution was added to each well and mixed. After 4 h, the supernatants were removed and 100 μL DMSO was added to each well to dissolve the precipitate. The cells viability was estimated by measuring absorbance at 570 nm using the MultiScan MCC 340 spectrophotometer (Thermo Fisher, Waltham, MA, USA). The IC50 values of CBL0137 on various cell lines were determined using Graph Pad Prism version 8.4.3. Results are presented as the mean ± standard deviation (SD).

### 2.3. Cell Cycle Analysis Using Flow Cytometer 

Cell cycle analysis was carried out according to PI staining protocol (Invitrogen, Waltham, MA, USA). Since cells at 24 h had different sensitivity to CBL0137, two concentration sets of CBL0137 were used (0.5, 0.75 and 1 µM and 1, 1.25 and 1.5 µM) for 24 h. Cells were collected, washed twice with ice-cold PBS and fixed with 70% ethanol at 4 °C 30 min. Then cells were stored at −20 °C for 24 h, then washed with PBS and stained with 0.02 mg/mL PI, 0.1% *v*/*v* Triton X-100 and 0.2 mg/mL DNase-free RNase A in PBS. After 30 min incubation at room temperature in the dark, cells were analyzed by flow cytometer FACSCalibur (Becton Dickinson, San Jose, CA, USA). Percent of cells in each cell cycle phase was analyzed and calculated using WinMDI software.

### 2.4. Annexin-FITC/Propidium Iodide Double Staining

Cells were stained with annexin V-FITC and PI to evaluate apoptosis by flow cytometry according to the manufacturer’s instructions to the FITC Anexin V Apoptosis Detection Kit I (Sigma-Aldrich, St. Louis, MI, USA). Briefly, cells were treated with 0.5–3 µM of CBL0137 for 24 h. After treatment, cells were collected, washed twice with ice-cold PBS, and resuspended in 0.5 mL of annexin/V-FITC/PI solution for 30 min in dark according to manufacturer protocol. After staining at room temperature, cells were analyzed by the flowcytometer FACSCalibur (Becton Dickinson, CA, USA). For each sample, 10,000 events were acquired and positive FITC and/or PI cells were quantified using WinMDI software.

### 2.5. RT2 Profiler PCR Arrays 

The Human Signal Transduction PathwayFinder™ RT2 Profiler™ PCR Array (PAHS-014Z, Qiagen, Frederick, MD, USA) was used to evaluate the expression of a panel of 84 genes representative of ten different signal transduction pathways, in FaDu cells treated with pepsin. Total RNA was cells with TRI reagent (Sigma-Aldrich, USA) in accordance with the manufacturer’s protocol. RNA was quantified using the Nanodrop 2000 (Gene Company Limited, Hong Kong, China), reverse transcription was performed using OT-1 reverse transcription kit (Synthol, Russia). First-strand cDNA was mixed with 2 × RT2 SYBR Green qPCR Master Mix and ddH2O. qPCR was performed in the Bio-Rad CFX96™ thermal cycler (Bio-Rad Laboratories, Hercules, CA, USA) using the following conditions: 95 °C for 10 min followed by the manufacturer’s protocol. Each array contained five independent housekeeping genes (Actb, B2m, Hprt1, Ldha and Rplp1) that were used for data normalization.

### 2.6. Experiments In Vivo 

Forty BALB/c male mice at age 8-10 weeks were obtained from the Stolbovaya farm and maintained in pathogen-free conditions. Experiments were carried out at the animal facility of the Blokhin National Medical Research Center of Oncology, Ministry of Health, Russian Federation. All experimental protocols were approved by the Ethics committee of our institution in accordance with national and international regulations (Resolution 81 of the Eurasian Economic Commission and directive 2010/63/EU) for the care and use of experimental animals (Blokhin Cancer Research Center, decision 2020-42 dated 27 October 2020). Mice were provided with autoclaved water and fed a standard powdered rodent diet ad libitum. Transplantable murine AML WEHI-3 was used to analyze anticancer activity of CBL0137 in vivo. Mice were randomized into five groups: two groups of 5 animals (group 1 and group 2) and three groups of 10 mice each (groups 3–5) were inoculated intraperitoneally by WEHI-3 cells. Eight days after inoculation of WEHI-3 cells, 10 mice of group 5 were treated every two days intravenously with CBL0137 (50 mg/kg). Animals from groups 1 and 3 groups were euthanized on the 8th day, and all other groups on the 22nd day. All organs were examined for gross pathology, and then liver and spleen were taken for histologic examination. Histologic analysis was performed on sections stained by hematoxylin-eosin. 

### 2.7. Data Processing 

All statistical analysis was carried out in GraphPad Prism 8.4.3 (La Jolla, CA, USA). The Kolmogorov–Smirnov (KS) test was used to determine the normality of the data. For normally distributed data (KS: *p* > 0.05), parametric tests such as the independent samples *t*-test and one-way analysis of variance (ANOVA) were used. For non-normally distributed data (KS: *p* < 0.05), the nonparametric Mann–Whitney U test or Kruskal–Wallis H-Test was used. The Pearson χ2 criterion was used to determine the statistical significance of differences in the analysis of the antitumor action of the drugs. Significance was set at *p* < 0.05 for all statistical analyses.

## 3. Results

### 3.1. Cytotoxic Effect of CBL0137

**Figure 1:**
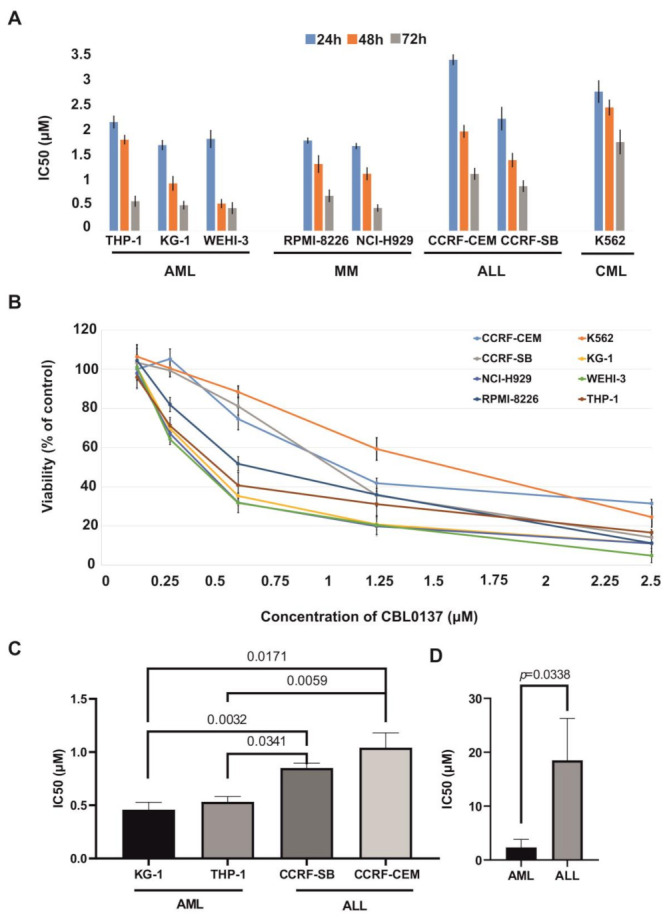
(**A**) IC50 after the cell treatment with CBL0137 over 24, 48 and 72 h; (**B**) cytotoxic effect of CBL0137 on hematological malignancy cells in vitro after 72 h treatment; (**C**) differences in the cytotoxic action CBL0137 among human cell lines of ALL and AML; (**D**) differences in the cytotoxic effects of CBL0137 on primary human ALL and AML cultures.

CBL0137 anticancer activity in vitro was studied in the human cell lines of hematological malignancies: KG-1 and THP-1 (acute myeloid leukemia, AML), CCRF-CEM and CCRF-SB (acute lymphocytic leukemia, ALL), K562 (chronic myeloid leukemia, CML), NCI-H929 and RPMI-8226 (multiple myeloma, MM) and murine AML cells WEHI-3. CBL0137 effects were assessed using the MTT assay after 24, 48 and 72 h of treatment (Figure 1A).

The IC50 after 72 h of CBL0137 treatment ranged from 0.41 to 1.60 μM. KG-1 (AML), NCI-H929 (MM) and WEHI-3 (murine AML) cells were the most sensitive to the cytotoxic activity of CBL0137, with IC50s of 0.47, 0.41 and 0.46 μM, respectively (Figure 1B,C). Statistically significant differences in CBL0137 IC50s after 72 h treatment were observed between AML cells (KG-1 and THP-1) and ALL cells; correspondingly, with CCRF-CEM, *p*-values were 0.0171 and 0.0032, and with CCRF-SB, *p*-values were 0.0341 and 0.0034. Similar statistically significant differences in CBL0137 IC50s were observed, when AML KG-1 and THP-1 were compared with CML K562 (corresponding *p*-values were 0.0132 and 0.0064). Cytotoxicity of CBL0137 against MM cells (NCI-H929) was also higher than against ALL cells (CCRF-CEM and CCRF-SB) and CML cells (K562), but significant differences were observed only between IC50s of MM and CML cells (corresponding *p*-values were 0.0059, 0.0222 and 0.0027). CBL0137’s cytotoxic activity against cells of myoblast primary cultures obtained from three AML patients were significantly more sensitive than lymphoblasts of two ALL patients (*p* = 0.0338) (Figure 1D). In addition, the IC50 value was not achieved when PBMC from healthy donors were treated with CBL0137, which indicates a low cytotoxic effect on normal blood cells (Figure S1).

### 3.2. Influence CBL0137 on the Cell Cycle Distribution

**Figure 2:**
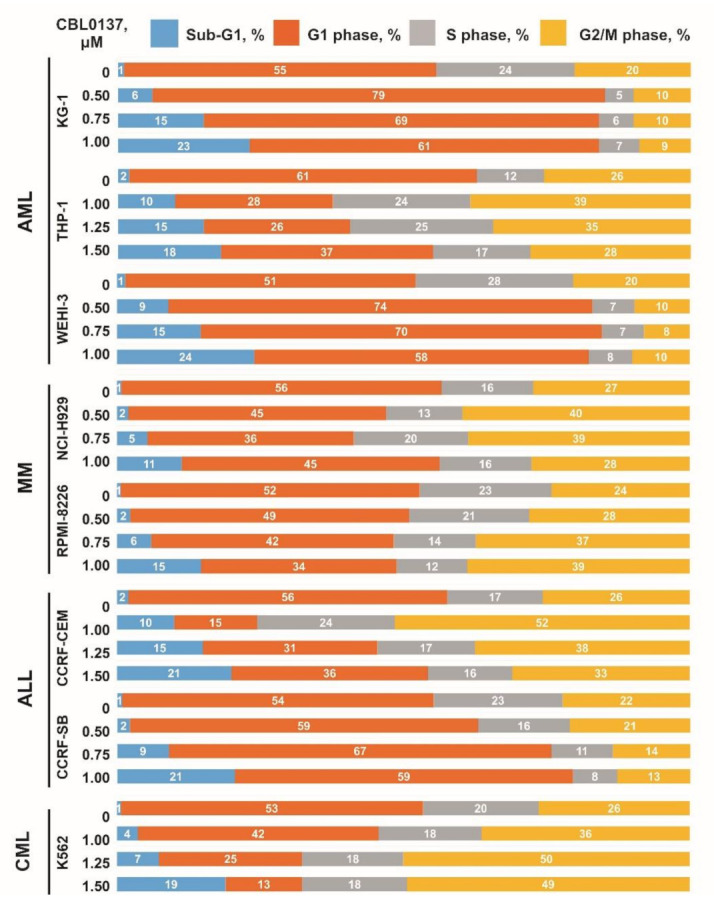
Effect of 24 h CBL0137 treatment on the distribution of cell cycle phases in cell populations.

Cell cycle analysis by flow cytofluorometry with propidium iodide (PI) was performed using CBL0137 concentrations that were less than 24 h IC30, as well as its ¾ and ½ dilutions. Accordingly, CBL0137 concentrations of 1.00, 0.75 and 0.5 μM were used to treat CCRF-SB, KG-1, WEHI-3, RPMI-8226 and NCI-H929 cells, and 1.50, 1.25 and 1.00 μM were used to treat CCRF-CEM, K562 and THP-1 (Figure S2). In the analysis of CBL0137 effects on cell cycle phase distribution, an increase in the sub-G1 cell population was observed in all the hematological malignancies cell lines. Simultaneously, a significant increase in the G1 phase cell population was observed in KG-1 and WEHI-3 AML cells, as well as CCRF-SB ALL cells, but it was not dose-dependent. We also revealed the cell cycle arrest of THP-1 AML cells, RPMI-8226, NCI-H929G2 MM cells, CCRF-CEM ALL cells and K562 CML cells in G2/M phase, caused by CBL0137 treatment (Figure 2 and Figure S2).

### 3.3. Influence CBL0137 on the Apoptosis Activation

**Figure 3:**
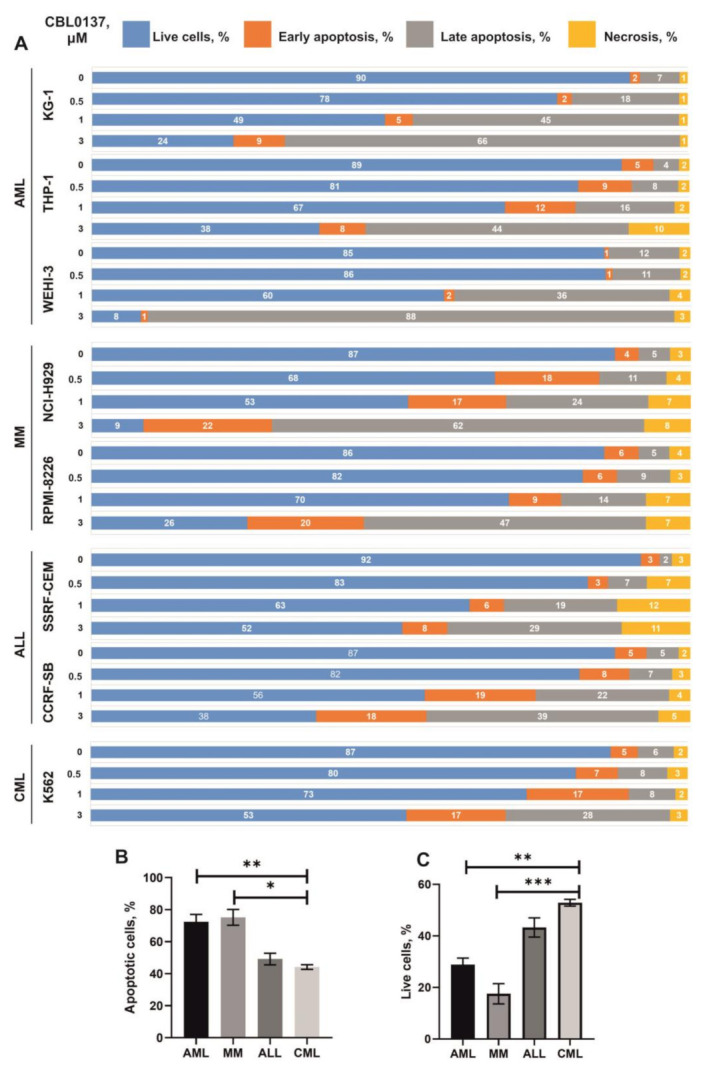
Effect of CBL0137 on the cell cycle in vitro after treatment within 24 h: (**A**) distribution of cell populations by fraction of living cells in necrosis and apoptosis (%); (**B**) proportions of populations of cells in apoptosis after treatment with 3 µM CBL0137; (**C**) proportion of living cells in the population after treatment with 3 µM CBL0137. * *p* ≤ 0.05, ** *p* ≤ 0.01, *** *p* ≤ 0.001.

Similar to the results obtained in the analysis of cell cycle distribution at IC30 and less, in all studied cells treated with 3 μM CBL0137 for 24 h and analyzed by flow cytometry using double staining with V-FITC Annexine and PI, we observed a dose-dependent increase in the sub-G1 peak, which is an indirect indicator of early-stage apoptosis. A dose-dependent increase in the proportion of cells with late apoptotic changes was also observed (Figure 3 and Figure S3). A statistically significant increase in the cell percentage with the induction of apoptosis was demonstrated in AML KG-1, THP-1 and WEHI-3 cell populations: we observed an increase in the portion of apoptotic cells from 13, 9 and 9% to 89, 59 and 75%, respectively. In MM cell lines NCI-H929 and RPMI08226, there was an increase in the proportion of apoptotic cells from 9% to 84% and from 11% to 67%, respectively. In CCRF-CEM and CCRF-SB cells, 24 h CBL0137 treatment increased the proportion of apoptotic cells up to 45% and 52% compared with 11% and 9% in the corresponding controls. CML cells K562 were the most resistant: the treatment induced an increase in the proportion of apoptotic cells from 5% to 37%.

### 3.4. Changes in the Activity of Signaling Pathways

Changes in the activity of signaling pathways in the THP-1, RPMI-8226, CCRF-CEM and CCRF-SB cell lines were analyzed with the Human Signal Transduction Pathway Finder RT2Profiler PCR Array set after a 24 h exposure to 1 μM CBL0137. A statistically significant decrease in the expression levels (more than 2-fold, *p* < 0.05) for 36 genes was observed in human AML THP-1, and an increase in the expression was identified for only the *FAS* gene of P53 signaling (Table 1). In RPMI-8226, expression level changes were identified for 38 genes; in particular, expression of 25 genes decreased and 13 genes increased. Expression changes in 43 genes were observed in CCRF-SB cells, including an expression decrease in 30 genes and an increase in 13 genes. In CCRF-CEM cells, expression changes were observed in 39 genes, of which 21 reduced their expression and 18 increased. In all the cells, the most significant changes were observed in the expression of the WNT and Hedgehog signaling pathway components. In THP-1, an intensive decrease was observed in the expression of all the studied WNT-associated genes; CCRF-SB, RPMI-8226 and CCRF-CEM cells showed a decrease in the expression of 7, 6 and 5 genes and an increase in the expression of 1, 1 and 2 genes, correspondingly. In THP-1 and CCRF-SB cells, we observed an intensive decrease in the expression of a number of Hedgehog signaling components, while an increase was not detected. Meanwhile, in CCRF-CEM and RPMI-8226 cells in Hedgehog signaling, we detected a decrease in expression in 5 and 6 genes and an increase in 1 and 2 genes, correspondingly (Table 1). 

### 3.5. Antileukemic Activity In Vivo

**Figure 4:**
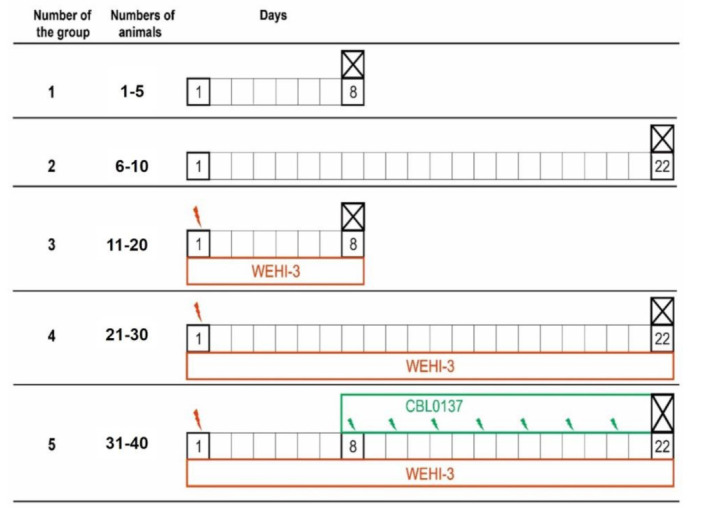
Experimental schedule for testing anticancer effect of CBL0137 on WEHI-3 in vivo. Mice were randomized into five groups: two groups of 5 animals (group 1 and group 2) and three groups of 10 mice each (groups 3–5) were inoculated intraperitoneally by WEHI-3 cells (brown arrows). Eight days after inoculation of WEHI-3 cells, 10 mice of group 5 were injected intravenously 50 mg/kg CBL0137 every two days (green arrows). Animals from groups 1 and 3 groups were euthanized on the 8th day and all other groups on the 22nd day.

Anticancer activity of CBL0137 in vivo using the murine AML model WEHI-3 developed over 50 years ago, which has been actively used in modern research to study anticancer effects of various plant compounds and extracts [14,99]. The experiment assessed the degree of pathological changes observed in the spleen and liver of BALB/C mice on the 7th day after inoculation with WEHI-3 tumor cells compared to control animals (before the treatment of CBL0137) and 22 days after inoculation with WEHI-3 tumor cells (after treatment with CBL0137 for 14 days) (Figure 4).

All animal body weights were measured at the end of the experiment; there were no statistical differences in this parameter among the groups. Liver and spleen tissues were isolated from all animals of each group. Spleen weights in the control group were significantly lower than in animals that were injected with Wehi-3 cells. CBL0137 treatment led to spleen weight reduction, but this difference was not statistically significant. Likewise, in liver weights, no significant differences among the groups were observed.

**Figure 5:**
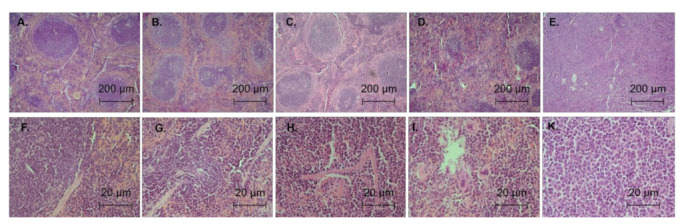
Microphotographs of spleen tissue with varying degrees of damage: (**A**,**F**)—0 (mice # 2); (**B**,**G**)—1 (mice # 15); (**C**,**H**)—2 (mice # 33); (**D**,**I**)—3 (mice # 27); (**E**,**K**)—4(mice # 29); (**A**–**E**)—x40, (**F**–**K**)—x400.

**Figure 6:**
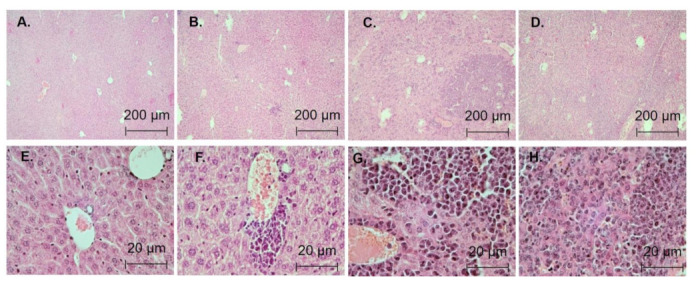
Microphotographs of liver tissue with varying degrees of damage: (**A**,**E**)—0 degree (mice # 2); (**B**,**F**)—1 degree (mice # 12); (**C**,**G**)—2 degree (mice # 22); (**D**,**H**)—3 degree (mice # 27); (**A**–**D**)—x40, (**E**–**H**)—x400.

The following criteria were used to analyze the degree of liver and spleen lesions (Table 2 and Table 3, Figure 5 and Figure 6): Seven days after inoculation with WEHI-3 cells, 80% of the animals had grade 1 spleen lesions and 20% of the animals had grade 2 lesions. Thus, at the beginning of CBL0137 treatment, 100% of the animals had leukemia. Moreover, 20% of the animals had small tumor infiltrates in liver parenchyma, corresponding to grade 1 liver lesions. In the 4th group, 22 days after the start of the experiment, we observed the following liver lesions: 20%—first degree, 20%—second, 40%—third and 20%—fourth (Table 4, Figure 5 and Figure 6). Liver damage was observed in 80% of animals: 10% first degree, 30% second degree and 40% total liver damage. In animals treated with CBL0137 (group 5) spleen lesions were found only in 50% of animals, with 10% of animals having the first degree lesion, 20% having the second and 20% having the third degree. The prevalence of liver damage in the 5th group was also decreasing. For example, only 40% of the animals had liver damage in this group, with 20% having the first degree of damage, and 10% having the second and third degrees of damage.

## 4. Discussion

The main obstacle to successful cancer chemotherapy is the genetic and epigenetic diversity of tumor clones, leading to the selection of resistant variants during treatment. In this regard, the proposed therapeutic drugs should be able to hit many targets and demonstrate a variety of mechanisms for inhibiting tumor growth. According to their estimated potential, each new item is investigated in the number of models in vitro and in vivo by using a range of putative targets. Following these requirements, we analyzed herein the anticancer effects of a small nongenotoxic DNA-binding molecule that was discovered as an inhibitor of NFkB and p53 activator with epigenetic chromatin remodulating activity. This is in accordance with current trend of epigenetic drug application for chemotherapy of hematological malignancies, and AML above all, as significant epigenetic dysregulation is considered to be a hallmark of this cancer type [13]. AML is the most lethal cancer among hematological malignances, and it is the main form of secondary cancers caused by anticancer therapy. It makes the search of new drugs against AML especially reasonable.

We showed that CBL0137 exerted a pronounced cytostatic effect in blood cancer cells, with IC50s in the range of 0.41 to 1.60 μM. This is in accordance with the study of Somers et al. 2020, who also demonstrated CBL0137’s cytotoxic effect in hematologic malignancy cells (IC50s were in the range of 0.41 to 0.9 μM) [12]. However, we focused our study on the comparison of CBL0137’s effects on the different types of hematological malignancies, and demonstrated for the first time the most pronounced cytotoxicity of CBL0137 against AML and MM. Thus, CBL0137 treatment over 72 h caused a higher cytostatic effect in AML (KG-1 and WEHI-3) and MM (NCI-H929) cells compared to ALL (CCRF-CEM) and CML (K562) cells. We also observed statistically significant differences in CBL0137 cytotoxic activity in primary cancer cells from the patients with AML and ALL.

In line with these results, we demonstrated more pronounced CBL0137-mediated apoptosis activation in AML (THP-1, KG-1 and WEHI-3) and MM (RPMI-8226 and NCI-H929) cells compared to the ALL (CCRF-CEM and CCRF-SB) and CML (K562) cells. In all studied cells, CBL0137 caused cell cycle arrest, and we observed a dose-dependent increase of sub-G1 both in AML and other forms of hematologic malignancies. Concurrently, CBL0137 induced cell cycle arrest in both the G1 or G2/M phase, and the prevalence of cell cycle arrest type varied independently on the hematological malignancy form. A similar effect of CBL0137 on the cell cycle was observed in medulloblastoma cell lines [100]. For example, J. Wang et al. demonstrated that CBL0137 treatment of the medulloblastoma cell line D458 resulted in accumulation of cells in the G1 phase, while treatment of HDMB03 cells with CBL0137 led to the prevalence of cells in the G2/M phase. In our study, in CCRF-SB cells, CBL0137 treatment caused cell cycle arrest in the G1 phase, which is probably associated with CDKN1B expression activation followed by the interruption of G1-S transition [101]. In addition, all investigated cells were characterized by decreased expression of CCND1 and CCND2 genes, which are the positive regulators of G1-S transition [102]. In THP-1, RPMI-8226 and CCRF-CEM cells, CBL0137 treatment reduced activity in signaling pathways inhibiting arrest in the G2/M phase, such as PPAR and WNT, which probably explains the observed cell cycle arrest in G2/M [103].

CBL0137 was discovered as a p53 activator, and when we analyzed its effects on cell signaling, the most intensive effect on p53 signaling was observed in AML cells [2]. We revealed an increase in the expression of *FAS* positively regulated by p53, and a significant decrease in the expression of *EGFR*, which is inhibited by p53 [84,85]. Thus, these data are in accordance with the results of Gasparian et al., who revealed CBL0137-mediated p53 activation and with the modern trend in AML chemotherapy to activate expression of tumor suppressors by epigenetic drugs [2,12].

The high anticancer potential of CBL0137 in all studied hematological malignancies is possibly related to the inhibition of WNT and Hedgehog signaling. Previously, gene cluster expression analysis demonstrated that the WNT and Hedgehog signaling pathways inhibit apoptosis and stimulate proliferation, overall disease progression and the reduction of therapy efficacy in hematological malignancy cells of different forms [104,105,106]. These signaling pathway components have been shown to cross-interact with each other and with signaling components involved in pathogenesis of hematological malignancies [107]. In particular, a positive regulator of WNT signaling transcription factor c-Myc induces Gli expression, which, in turn, activates Hedgehog signaling [108]. CBL0137 was shown to reduce c-Myc expression, thereby inhibiting WNT signaling activity [11]. Thus, inhibition of Hedgehog and WNT signaling in the cells of all studied hematological malignancies may be associated, at least partially, with the decreased c-Myc expression.

Further, we observed Notch signaling activation in the ALL cell lines, with the most pronounced effect in CCRF-CEM cells (an activation of 8 out of 9 genes with changed expression) and in the CCRF-SB cells (3 out of 9 genes with changed expression), while in AML THP-1 cells, several components of Notch signaling were decreased, and no one was activated. Early activation of Notch signaling by CBL0137 has been previously shown in other types of cancers, in particular, small cell lung cancer cells [8]. However, it should be noted that Notch signaling activation in tumor cells could be associated both with tumor progression and suppression [109]. For example, in small cell lung cancer, Notch signaling is inactivated and Notch activation leads to tumor cell death [110], while Notch hyperactivation was shown in more than 50% of ALL cases, and it afforded tumor cells to escape from p53-mediated apoptosis [111,112]. Thus, the observed difference in CBL0137 effects in AML and ALL cells may be also mediated by Notch signaling activation in ALL.

Our data are in accordance with the study that reported anticancer effects of thymoquinone on WEHI-3 cells in vitro and in vivo [14]. Thymoquinone is a plant secondary metabolite with epigenetic activity. It modifies histone acetylation and deacetylation, DNA methylation and demethylation and alters the genetic expression of various noncoding RNAs [113]. It caused in WEHI-3 cells cell cycle arrest in G1 phase and induced apoptosis. The ability of thymoquinone to defend in vivo murine liver and spleen tissues morphology from destroying by leukemia cells was similar to that by Curaxin CBL0137, which is also epigenetically active agent.

## 5. Conclusions

In the presented study, we demonstrated that CBL0137 is effective in cell lines and primary cultures of hematological malignancies by triggering apoptosis and inhibiting cell cycle progression, as well as suppressing the development of in vivo tumors. CBL0137 suppresses the survival and developmental pathways such as WNT and Hedgehog in tumor cells. Of particular importance are the data that we have obtained on the most pronounced effects of CBL0137 in human in vitro cultured AML and MM cells and in WEHI-3 in vivo mice AML. This is due to the fact that despite the success of modern chemotherapy, the 5-year overall survival rate for AML is only 28% [114], and MM continues to be one of the most common forms of hematological malignancies [115]. These findings suggest that CBL0137 may be an effective treatment for these types of hematological malignancies.

## Figures and Tables

**Table 1:** Effect of CBL0137 on cell signaling pathways in AML, MM and ALL cell lines.

Gene		Function											Log_2_ (Expression Fold Change)					
													THP-1	RPMI-8226	CCRF-SB		CCRF-CEM	
WNT signaling																		
AXIN2		Negative regulator of WNT, expression activated by β-catenin [15]											−3.5	−2.4	−2.5		−10.5	
CCND1		Activates the transition from G1 to S phase of the cell cycle. target gene of WNT pathway [15]											*	−2.1	−2.9		−3.4	
CCND2		Activates the transition from G1 to S phase of the cell cycle. target gene of WNT pathway [16]											−4.0	−2.7	−2.8		−5.0	
FOSL1		Positive regulator of proliferation, target gene for WNT pathway [17]											*	*	2.1		5.1	
MMP7		Regulates remodeling extracellular matrix, positive regulator of EMP, target gene of WNT pathway [18]											−5.9	−2.5	−2.7		−2.3	
MYC		Transcription factor, positive WNT pathway regulator [19]											−4.3	−2.7	−3.5		−5.8	
WISP		Positive WNT [20] pathway regulator, expression corresponds to pathway activity [20]											−9.8	3.1	3.6		2.9	
PPARD		Positive WNT signaling regulator [21]											−2.9	*	−2.5		*	
DAB2		Negative regulator of the WNT receptor [16]											−3.2	−2.4	*		*	
Notch signaling																		
HES5		Notch pathway target gene [22], negative regulator of cell differentiation [23]											*	*	2.6		4.0	
HEY1		Notch pathway target gene [24], positive regulator of cell differentiation [25]											−5.7	*	2.0		6.7	
HEY2		Notch pathway target gene [26], positive regulator of cell differentiation [27]											−8.1	*	*		3.9	
HEYL		Notch pathway target gene [28], positive regulator of cell differentiation [29]											*	*	*		4.9	
ID1		Negative regulator of Notch pathway [30], expression dependent on pathway activity [31]											−6.6	3.6	2.8		6	
JAG1		Positive regulator of Notch pathway [32]											*	*	*		7.8	
LFNG		Positive regulator of Notch pathway [33]											*	*	*		3.3	
NOTCH1		Positive regulator of Notch pathway [34]											*	*	*		4.4	
Hedgehog-signaling																		
BCL2		Target gene of Hedgehog pathway, negative regulator of apoptosis [35]											*	−2.7	−2.0		−2.9	
PTCH1		Negative regulator of Hedgehog pathway [36], expression dependent on pathway activity [37]											*	2.7	−3.0		4.3	
BMP4		Positive regulator of the Hedgehog pathway [38], expression depends on the activity of the pathway [39]											−4.5	*	−3.5		*	
WNT1		Hedgehog pathway target gene [40], positive regulator of WNT pathway											−5	−3.1	*		−5.8	
WNT2B		Hedgehog pathway target gene [40], positive regulator of WNT pathway											−3.1	−5.1	−2.9		−4.5	
WNT3A		Hedgehog pathway target gene [40], positive regulator of WNT pathway											*	*	*		−4.8	
WNT5A		Hedgehog pathway target gene [40], positive regulator of WNT pathway											−13.9	−2.5	−2.4		−5.7	
WNT6		Hedgehog pathway target gene [40], positive regulator of WNT pathway											−4.8	−2.4	*		*	
Hypoxia-induced signaling																		
ADM		HiF-1/HiF-2-dependent gene [41], vasodilator hormone. angiogenesis stimulant [42]											−2.9	*	2.5		*	
CA9		HiF-1-dependent gene [43], catalyzes CO2 hydration, trunk maintenance [44]											−13.3	−2.5	*		−2.3	
EPO		HiF-1-dependent gene [45], regulates erythropoiesis and angiogenesis [46]											−15.5	2.3	*		*	
HMOX1		HiF-1-dependent gene, involved in heme metabolism and response to oxidative stress [47]											−2.5	*	*		*	
SERPINE1		HiF-1-dependent gene [48]. activator of hemostasis, cellular migration, angiogenesis [49]											−6.5	−3.3	*		*	
SLC2A1		HiF-1-dependent gene [50], transports glucose to the cell [51]											−3.2	2.3	−2.6		*	
VEGFA		HiF-1-dependent gene [52], positive regulator of angiogenesis [53]											−2.9	−2.7	−3.2		−5.3	
LDH1		Positive regulator of HiF-1 expression [54]											*	−2.3	−2.5		*	
NF-κΒ signaling																		
CSF1		Genomic target of NF-κΒ pathway [55], acts as a stimulant of proliferation [56]											−2.9	*	*		*	
IFNG		The target gene that positively regulates the immune response [57]											−4.4	−2.6	3.2		5.9	
BCL2A1		A target gene of the signaling pathway [58], negatively regulates apoptosis [59]											*	−3.0	*		*	
BIRC3		A target gene of the signaling pathway [60], negatively regulates apoptosis [61]											*	*	−5.3		−3.3	
TNF		A positive regulator of the NF-kB pathway [62], expression is dependent on the activity of the pathway [63]											*	−3.0	−2.4		3.2	
STAT1		Is a positive regulator of the NF-kB signaling pathway [64]											*	6.0	*		*	
CCL5		A target gene of pathway [65], positively regulates the immune response [66]											*	*	*		2.9	
TGFβ signaling pathway																		
ATF4		Target gene of TGFβ pathway, promotes stem-like phenotype in tumor cells [67]											−3.5	−2.8	−2.1		*	
EMP1		Target gene of TGFβ pathway, activates proliferation and cellular migration [68]											−4.1	*	−3.9		*	
MYC		Transcription factor, negative regulator of TGFβ [69]											−4.3	−2.7	−3.5		−5.8	
HERPUD1		TGFβ target gene, component of ER-dependent degradation system [70]											*	−5	−2.2		*	
GADD45B		Target gene of TGFβ pathway, negatively regulates apoptosis, activates repair [71]											*	3.2	−7.9		*	
IFRD1		Target gene of TGFβ pathway, regulates immune response [72]											*	2.3	−2.4		−2.0	
CDKN1B		Target gene of TGFβ pathway [73], negatively regulates cell cycle progression [74]											*	*	2.1		*	
TNFSF10		Target gene in TGFβ pathway [75], activates apoptosis [76]											*	*			6.4	
PPARγ signaling pathway																		
CPT2		PPARγ target gene, fatty acid oxidation enzyme [77]											−3.2	2.7	−2.4		*	
FABP1		PPARγ target gene, transport of fatty acids [77]											−6.6	*	*		*	
OLR1		PPARγ target gene, mediates endocytosis of low-density lipoproteins [78]											−7.1	−3.5	−2.6		3.8	
SLC27A4		PPARγ target gene, mediates the transport of fatty acids inside the cell [79]											−4.6	2.4	*		*	
SOCS3		PPARγ target gene, negative regulator via JAK/STAT [80]											−2.1	*	2.0		3.2	
ACSL3		Target gene of PPARγ-pathway, mediates acyl-CoA joining to fatty acids [81]											*	2.3	−2.5		*	
ACSL4		Target gene of PPARγ-pathway, mediates acyl-CoA joining to fatty acids [82]											*	*	−2.4		*	
ACSL5		Target gene PPARγ pathways, mediates acyl-CoA joining to fatty acids. [77]											*	*	−2.1		*	
P53 signaling pathway																		
BBC3		Target gene of P53, activates apoptosis [83]											−3.2	−2.8	2.7		*	
EGFR		Expression mediated by p53 interaction with miR27a, tyrosine kinase receptor [84]											−23.0	*	−3.9		−3.9	
FAS		Target gene of P53, activates external apoptosis pathway [85]											2.4	*	−2.4		*	
BTG2		Target gene of P53, inhibits cell cycle progression [86]											*	−3.4	*		*	
PCNA		Target gene of P53, increases DNA polymerase efficiency [87]											*	2.0	−2.4		*	
BAX		Target gene of P53, activates apoptosis [88]											*	*	*		−5.0	
JAK/STAT signaling pathway																		
SOCS3		Negative regulator of JAK/STAT pathway [89]											−2.1	*	2.0		3.0	
FCER2		Target gene of JAK/STAT pathway, stimulates growth and differentiation of B cells [90]											−16.6	*	−4.4		*	
CCND1		Activates the transition from G1 to S phase of the cell cycle, target gene of the JAK/STAT pathway [91]											*	−2.2	−2.0		−3.4	
IRF1		Receptor that activates the JAK/STAT signaling pathway [92]											*	*	*		−7.6	
CEBPD		Negative regulator of the JAK/STAT signaling pathway, induces cell arrest and apoptosis initiation [93]											*	*	2.5		*	
LRG1		Positive JAK/STAT pathway regulator, suppresses apoptosis and induces G1 phase passage [94]											*	*	2.6		*	
Oxidative stress signaling pathway																		
GSR		Target gene of the pathway, reduces oxidized glutathione disulfide [95]											−3.3	*	*		*	
HMOX1		Target gene of the pathway, activates heme metabolism [96]											−2.5	*	*		*	
TXNRD1		Target gene of the signaling pathway, protection against oxidative stress [97]											*	2.4	2.4		*	
NQO1		Target gene of the signaling pathway, protection against oxidative stress (quinone reduction) [98]											*	*	*		−2.5	
Log_2_(fold change)																		
−10.0	−9.0	−8.0	−7.0	−6.0	−5.0	−4.0	−3.0	−2.0		2.0	3.0	4.0	5.0	6.0	7.0	8.0	9.0	10.0

*—Log2 fold change is in the interval (−2.0; +2.0).

**Table 2:** Morphologic criteria for the degree of spleen lesions caused by WEHI-3 leukemia.

Degree of Damage	Changes in Spleen Structure
Stage 0	Intact organ
Stage 1	The nodules of the white pulp are preserved, distinct contours are observed, and the structure with hermial centers is intact. The red pulp contains megacaryocytes (as a marker of hematopoiesis) and single tumor cells
Stage 2	There is a change in the structure of the white pulp, but with distinct contours. Germinal centers are observed only in half of nodules. The red pulp contains groups of tumor cells and megacaryocytes
Stage 3	The number of nodules of the white pulp is reduced to 2 or less in the field of view; the structure of nodules is changed. The red pulp is infiltrated by tumor cells, and no hemopoiesis has been observed
Stage 4	The cytoarchitectonics of the spleen is broken, the boundary between the red and white pulp is missing, and there is an infiltration of tumor cells throughout

**Table 3:** Morphologic criteria for the degree of liver lesions caused by WEHI-3 leukemia.

Degree of Damage	Changes in Organ Structure
Stage 0	Intact organ
Stage 1	Single tumor cells or small infiltrates
Stage 2	Medium-sized tumor infiltrators, large-scale tumor infiltrates
Stage 3	Large tumor infiltrates, liver lesions destroying cytoarchitectonics

**Table 4:** Effect of CBL0137 on the degree of leukemia-induced damage to the spleen and liver of mice in the development of transmissible leukemia WEHI-3.

Degree of Damage	Control Mice (7 Days)	WEHI-3 (7 Days)	Control Mice (21 Days)	WEHI-3 (21 Days)	WEHI-3 (21 Days) + CBL0137 (7–21 Days)
Spleens					
0	5/5	0/10	5/5	0/10	5/10
1	0/5	8/10	0/5	2/10	1/10
2	0/5	2/10	0/5	2/10	2/10
3	0/5	0/10	0/5	4/10	2/10
4	0/5	0/10	0/5	2/10	0/10
Livers					
0	5/5	8/10	5/5	2/10	6/10
1	0/5	2/10	0/5	1/10	2/10
3	0/5	0/10	0/5	3/10	1/10
4	0/5	0/10	0/5	4/10	1/10

## Data Availability

Not applicable.

## Supplementary Materials

The following supporting information can be downloaded at: https://www.mdpi.com/article/10.3390/biomedicines11010230/s1, Figure S1: Comparison of the CBL0137’s cytotoxic effect on PBMC, AML and ALL cells culturest, Figure S2: An example of the CBL0137’s effect on the distribution of the cell cycle in hematologic malignancies cultures, Figure S3: An example of the CBL0137’s effect on the activation of apoptosis in in hematologic malignancies cultures.
