# Supplementary material for: Targeting Features of Curaxin CBL0137 on Hematological Malignancies In Vitro and In Vivo
Source: Biomedicines. 2023 Jan 16;11(1):230. doi: 10.3390/biomedicines11010230 (PMC9856019; doi:10.3390/biomedicines11010230)
Supplement: Supplementary file 1 [file biomedicines-11-00230-s001.zip › biomedicines-2102456-supplementary.pdf]

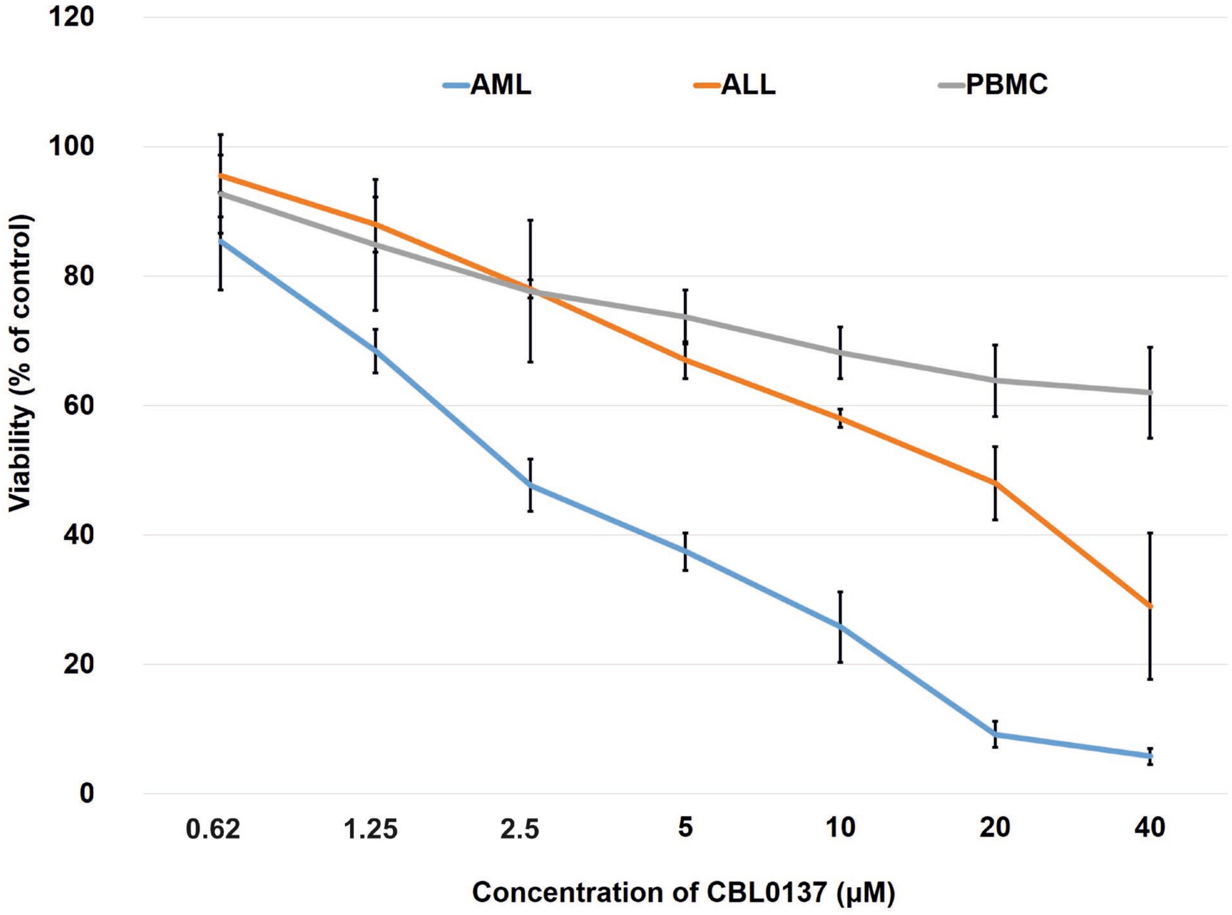

Figure S1. Comparison of the CBL0137's cytotoxic effect on PBMC, AML and ALL cells cultures

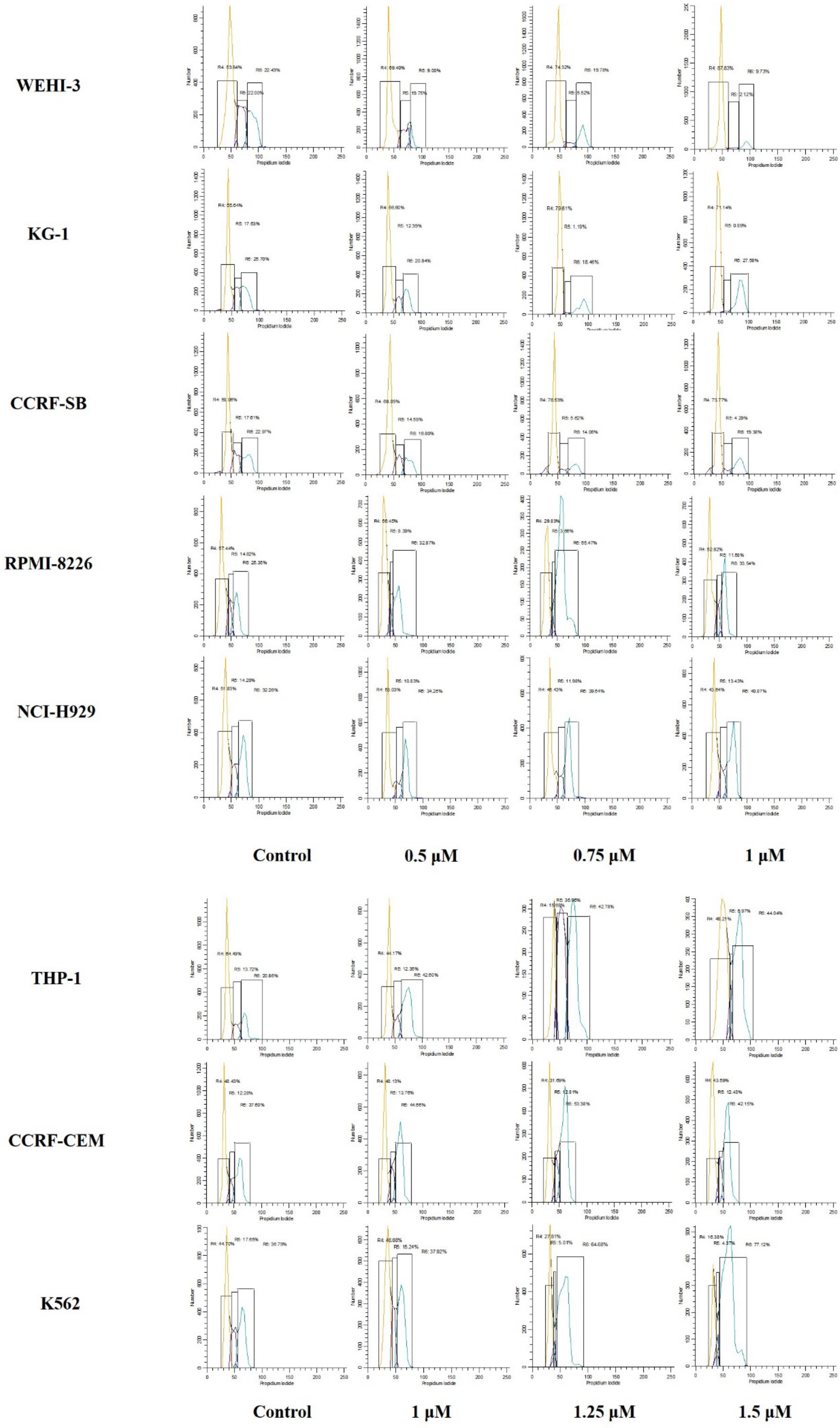

Figure S2. An example of the CBL0137's effect on the distribution of the cell cycle in hematologic malignancies cultures

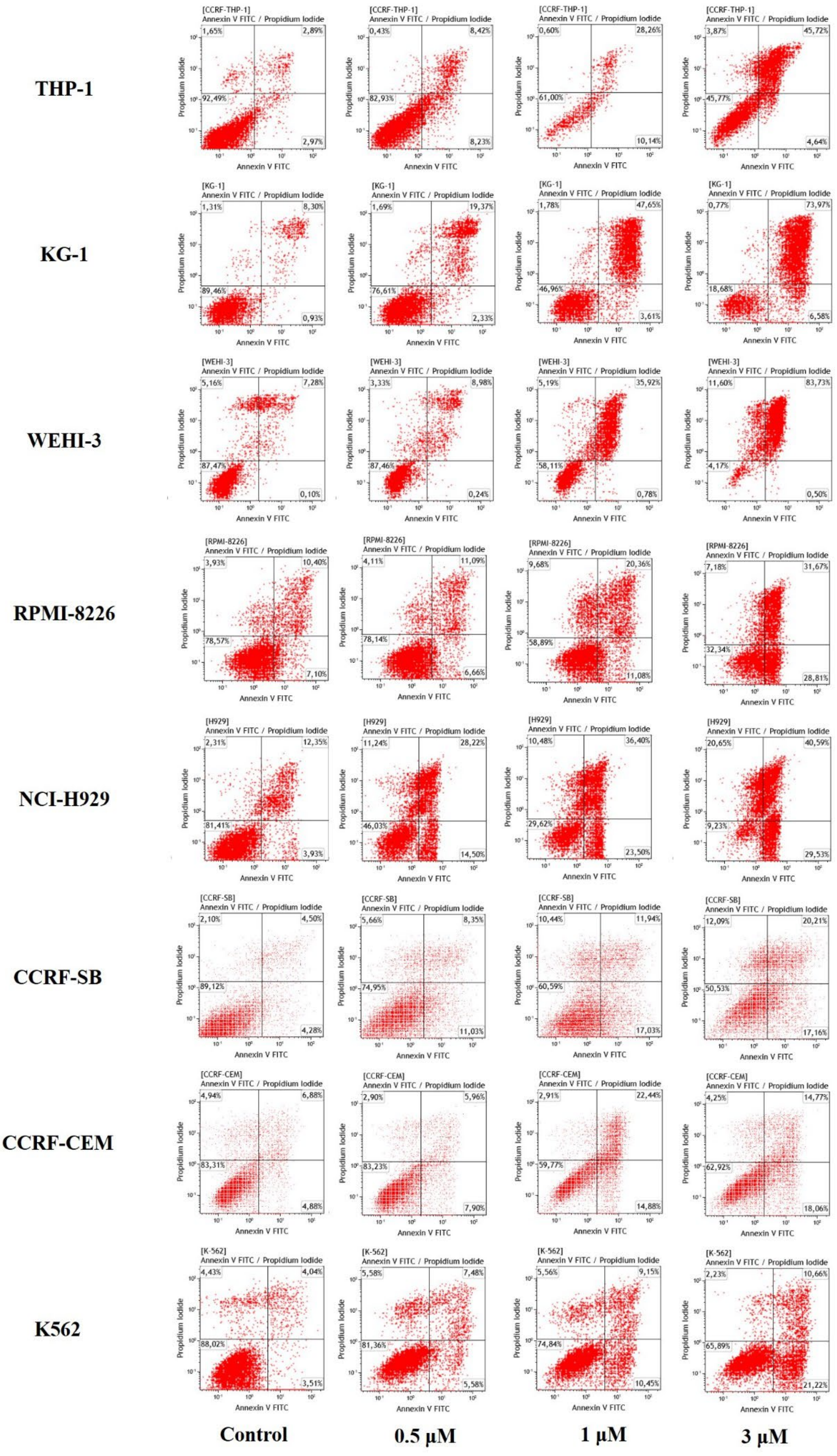

Figure S3. An example of the CBL0137's effect on the activation of apoptosis in in hematologic malignancies cultures
